# Marked Seasonal Variation in Structure and Function of Gut Microbiota in Forest and Alpine Musk Deer

**DOI:** 10.3389/fmicb.2021.699797

**Published:** 2021-09-06

**Authors:** Feng Jiang, Hongmei Gao, Wen Qin, Pengfei Song, Haijing Wang, Jingjie Zhang, Daoxin Liu, Dong Wang, Tongzuo Zhang

**Affiliations:** ^1^Key Laboratory of Adaptation and Evolution of Plateau Biota, Northwest Institute of Plateau Biology, Chinese Academy of Sciences (CAS), Xining, China; ^2^College of Life Sciences, University of Chinese Academy of Sciences, Beijing, China; ^3^Qinghai Provincial Key Laboratory of Animal Ecological Genomics, Xining, China; ^4^State Key Laboratory of Plateau Ecology and Agriculture, Qinghai University, Xining, China

**Keywords:** musk deer, 16S rRNA gene sequencing, seasonal variation, core microbiome, metabolic functions

## Abstract

Musk deer (*Moschus* spp.) is a globally endangered species due to excessive hunting and habitat fragmentation. Captive breeding of musk deer can efficiently relieve the hunting pressure and contribute to the conservation of the wild population and musk supply. However, its effect on the gut microbiota of musk deer is unclear. Recent studies have indicated that gut microbiota is associated with host health and its environmental adaption, influenced by many factors. Herein, high-throughput sequencing of the 16S rRNA gene was used based on 262 fecal samples from forest musk deer (*M. berezovskii*) (FMD) and 90 samples from alpine musk deer (*M. chrysogaster*) (AMD). We sought to determine whether seasonal variation can affect the structure and function of gut microbiota in musk deer. The results demonstrated that FMD and AMD had higher α-diversity of gut microbiota in the cold season than in the warm season, suggesting that season change can affect gut microbiota diversity in musk deer. Principal coordinate analysis (PCoA) also revealed significant seasonal differences in the structure and function of gut microbiota in AMD and FMD. Particularly, phyla *Firmicutes* and *Bacteroidetes* significantly dominated the 352 fecal samples from captive FMD and AMD. The relative abundance of *Firmicutes* and the ratio of *Firmicutes* to *Bacteroidetes* were significantly decreased in summer than in spring and substantially increased in winter than in summer. In contrast, the relative abundance of *Bacteroidetes* showed opposite results. Furthermore, dominant bacterial genera and main metabolic functions of gut microbiota in musk deer showed significant seasonal differences. Overall, the abundance of main gut microbiota metabolic functions in FMD was significantly higher in the cold season. WGCNA analysis indicated that OTU6606, OTU5027, OTU7522, and OTU3787 were at the core of the network and significantly related with the seasonal variation. These results indicated that the structure and function in the gut microbiota of captive musk deer vary with seasons, which is beneficial to the environmental adaptation and the digestion and metabolism of food. This study provides valuable insights into the healthy captive breeding of musk deer and future reintroduction programs to recover wild populations.

## Introduction

Musk deer (*Moschus* spp.), the only extant genus of the family Moschidae, consists of seven species and are widely found in forests and mountains in Asia (Yang et al., 2003; Jiang et al., 2020). China has the largest musk deer population and musk source globally (Sun et al., 2018). Six species of genus *Moschus* are found in China, among which the forest musk deer (FMD, *M. berezovskii*) and the alpine musk deer (AMD, *M. chrysogaster*) are the most widely distributed and have the highest wild and captive population (Fan et al., 2018). They inhabit high-altitude coniferous forests or broad-leaved forests (Wang and Harris, 2015), alpine shrub meadow, and mountain forest grassland zone (Harris, 2016) in central and southwestern China, with some overlapping areas. However, wild musk deer populations plummeted to the brink of extinction in the 20th century due to illegal hunting, habitat fragmentation, and other human activities (Wu and Wang, 2006; Cai et al., 2020). The International Union for Conservation of Nature (IUCN) Red List (Wang and Harris, 2015; Harris, 2016) and red list of China’s vertebrates (Jiang et al., 2016) have listed both the species as endangered (EN) and critically endangered (CR), respectively. The captivity breeding of the FMD and AMD began in China in the 1960s to curb the rapid decline of the musk deer population by reducing the pressure on hunting wild musk deer to some extent (Fan et al., 2019). Captive individuals can also serve as a rewilding resource for reintroduction, which is beneficial for effective conservation and population recovery of wild musk deer. However, gastrointestinal diseases occur in the captive FMD and AMD with a fatality rate of about 30%, especially in winter and autumn (Li et al., 2017). Diseases are the most significant constraints on population growth, breeding scale, and musk secretion (Zhao et al., 2011; Zhang et al., 2019).

Gut microbiota has a close mutualistic symbiotic relationship with their hosts during long-term coevolution and is essential in organisms (Nicholson et al., 2012). The complex and variable micro-ecosystem of gut microorganisms were involved in metabolism, immune regulation, intestinal development promotion, pathogen defense, and other physiological activities (Wang et al., 2019; Yoo et al., 2020). Many factors, such as host genetics, diet, season, age, and lifestyle, influence the composition and function of gut microbiota (Zhang et al., 2010; Claesson et al., 2012; O’Toole and Jeffery, 2015). For instance, changes in dietary composition rapidly alter the composition and abundance of gut microbiota (Zmora et al., 2019). Seasonal changes in food composition and availability change the structure and function of gut microbiota in many animals (Amato et al., 2015; Xue et al., 2015). Moreover, captive breeding and *ex situ* conservation are effective for the conservation of endangered species. Long-term captive breeding has significantly changed the dietary composition of musk deer compared with wild populations (Guo et al., 2019), resulting in changes in the composition and function of gut microbiota (Sun et al., 2020). Therefore, studying the diversity of gut microbiota of endangered species in captivity is essential for assessing the current captive conditions, understanding the appropriate capacity of changes in gut microbiota for their future, and evaluating whether they can be released into the wild. However, the relationship between the diversity, structure, and function of gut microbiota of captive musk deer of different ages and seasonal changes is unclear due to the inadequate relevant data.

In this study, combined with weighted gene co-expression network analysis (WGCNA), the 16S rRNA gene amplicon technology was used for high-throughput sequencing on the Illumina MiSeq sequencing platform to analyze the fecal microbial composition, diversity, and function in captive FMD and AMD in different seasons. This study aimed to (i) explore the composition and differences in the gut microbiota of both musk deer in different seasons; (ii) analyze the core microbiota and its metabolic functions, and their seasonal difference; and (iii) construct a weighed co-occurrence network of gut microbiota for identifying modules of co-occurring taxa and hub OTUs significantly related with the seasonal variation. Therefore, this study can provide a scientific basis for the effective management of captive musk deer.

## Materials and Methods

### Sample Collection

A non-invasive sampling method was used to collect 262 fresh feces of captive FMD in Aru Township, Qilian County, Qinghai Province, China (100°21′E, 38°7′N), during the early spring (mid-March, here referred to as T1, *N* = 49), late spring (late May, here referred to as T2, *N* = 57), summer (mid-July, here referred to as T3, *N* = 56), autumn (mid-November, here referred to as T4, *N* = 50), and winter (late December, here referred to as T5, *N* = 50). The FMD breeding center was located northeast of the Qinghai–Tibet Plateau with an altitude, annual average temperature, and precipitation of about 3,002 m, −0.1°C, and 403 mm, respectively, with high daily range and radiation intensity. The maximum and minimum temperatures appeared in July and January, respectively. Furthermore, 90 fecal samples of captive AMD were collected during the late spring (late May, here referred to as T2, *N* = 55) and winter (late December, here referred to as T5, *N* = 35) in Xinglong Mountain, Gansu Province (104°4′E, 35°49′N). The AMD breeding center was located northeast of the Qinghai–Tibet Plateau with an altitude, annual average temperature, and precipitation of 2,171 m, 5.4°C, and 406 mm, respectively.

The FMD and AMD breeding centers were cleaned the night before sampling, and each individual was kept in a separate enclosure so that the fresh feces of each individual could be collected the following morning. Disposable PE gloves were used to collect fresh feces and put them into sterile self-sealed bags to prevent contamination of the feces surface. All samples were temporarily stored in the −20°C vehicle-mounted refrigerator and later stored in the −80°C ultra-low temperature refrigerator in the laboratory for DNA extraction.

### DNA Extraction and 16S rRNA Gene Sequencing

E.Z.N.A.^®^ soil DNA Kit (Omega Biotek, Norcross, GA, United States) was used to extract total bacterial DNA from FMD and AMD feces. The extraction quality of DNA was assessed using 1% agarose gel electrophoresis. NanoDrop 2000 (Thermo Fisher Scientific, Waltham, MA, United States) was used to determine the concentration and purity of DNA. The V4–V5 variable region of the 16S rRNA gene was amplified *via* PCR using primers; 515F (5′-GTGCCAGCMGCCGCGG-3′) and 907R (5′-CCGTCAATTCMTTTRAGTTT-3′). Each sample had three PCR replicates.

The PCR was run in a reaction volume of 20 μl, containing 4 μl TransStart FastPfu buffer (5×), 2 μl dNTPs (2.5 mM), 0.8 μl each of forward and reverse primers (5 μM), 0.4 μl TransStart FastPfu DNA Polymerase, and 10 ng sample DNA and topped up with ddH_2_O. Throughout the PCR amplification, ultrapure water was used instead of a sample solution as a negative control to eliminate the possibility of false-positive PCR results. The PCR amplification procedure was as follows: 95°C for 3 min (initial denaturation), followed by 27 cycles at 95°C for 30 s (denaturing), 55°C for 30 s (annealing), and 72°C for 45 s (extension), and a final single extension at 72°C for 10 min.

The same sample PCR products were mixed, then 2% agarose gel was used to recover the PCR products. AxyPrep DNA Gel Extraction Kit (Axygen Biosciences, Union City, CA, United States) was used to purify the recovered products. The recovered products were measured using 2% agarose gel electrophoresis and quantified using Quantus^TM^ Fluorometer (Promega, United States). NEXTFLEX Rapid DNA-Seq Kit (Bioo Scientific, Austin, TX, United States) was used to build the library according to the manufacturer’s protocols. Then, the purified PCR-amplified fragments were pooled in equal concentrations and sequenced on Illumina MiSeq PE300 platform (San Diego, CA, United States).

### Determination of OTU and Taxonomy Assignments

The raw data were pre-processed to remove the known adaptor, specific primers and low-quality ends with Trimmomatic (version 0.39, PE-phred33 ILLUMINACLIP:2:30:10 TRAILING:20 MINLEN:50 SLIDINGWINDOW:50:20) (Bolger et al., 2014). We set a 50-bp slide window and trimmed off those sequences with average base quality <20. These low-quality reads include reads with >10 nucleotides aligned to the adapter sequences, those with unidentified nucleotide (N) sequences, and those read lengths below 50 bp. The generated forward and reverse unpaired sequences were than merged together using FLASH with a minimum overlap of 10 bp and maximum mismatch of 0.2 (version 1.2.7, -m 10 -x 0.2) (Magoč and Salzberg, 2011).

Chimera reads were removed, and operational taxonomic units (OTUs) were clustered with 97% nucleotide sequence similarity using UPARSE (version 7.1)^1^ (Costello et al., 2009). The highest-frequency sequence in each OTU was selected as the representative sequence for further annotation. The reference sequence annotation and curation pipeline (RESCRIPt) was used to prepare a compatible Silva 138/16s bacteria database, based on SILVA SSURef of the curated NR99 (version 138) database^2^ followed the protocol suggested by author^3^. The Ribosomal Database Project (RDP) classifier (version 2.11)^4^ was used to classify the representative OTU sequence against the Silva 138/16s bacteria database at a confidence threshold of 0.8 (Li et al., 2017). The taxonomy-based filtering was applied to remove all features that contain either mitochondria, chloroplast, or archaea, and the results were aligned to generate the OTU table.

### Bioinformatic Analysis

Operational taxonomic units that were present in any two samples that are less than five sequences as well as a total abundance (summed across all samples) of less than 10 also be filtered. The OTU table was than rarefied to the lowest number of reads across samples (for FMD of 49,615 and for AMD of 50,680) before downstream analysis. The corresponding abundance information of each OTU annotation result in each sample was counted, and the sample sequences were defined as OTU based on the minimum number of sample sequences. Community bar charts were used to plot the relative abundance of bacteria in each fecal sample in musk deer at phylum and genus levels with R software (version 3.3.1, packages “stats”). Venn charts were used to analyze the core and unique bacterial phyla and genera in different seasons with the R software. A cluster heat map was used to compare the composition in different seasons with the R software (packages “pheatmap”) (Perry, 2016). Alpha diversity was used to reflect the diversity of intestinal microbial composition. Sobs index (the observed richness) and Shannon index were used to analyze the diversity of gut microbial composition at the OTU level using Qiime software^5^ (Caporaso et al., 2010). The Wilcoxon rank-sum test was used to analyze the seasonal significance of the two Alpha diversity indexes with the R software (packages “stats”).

The bacterial diversity among different microbial communities was compared and analyzed to explore the community composition among different seasonal groups. Principal coordinate analysis (PCoA) was used to analyze the beta diversity among different groups with the R software (packages “vegan”). Bray–Curtis was used to calculate the distance between samples at the OTU level. Analysis of similarities (ANOSIM), a non-parametric statistical test, was used for the intergroup difference test with the R software (packages “vegan,” anosim function) (Oksanen et al., 2019). The metabolic functions of bacterial communities were predicted using PICRUSt (Phylogenetic Investigation of Communities by Reconstruction of Unobserved States) software based on the KEGG (Kyoto Encyclopedia of Genes and Genomes) and EggNOG (Evolutionary Genealogy of Genes: Non-supervised Orthologous Groups) databases with the OTU species annotation and abundance information (Langille et al., 2013). Moreover, the Wilcoxon rank-sum test was used to analyze the seasonal differences of metabolism-related functions and dominant bacteria in all groups.

Weighted gene co-expression network analysis is a system biology method originally conceived for describing correlation patterns among genes across microarray data, which is widely used to identify critical hub genes of biological processes by constructing gene co-expression networks (Saris et al., 2009). Currently, it has been also applied in gastric microbiome networks and microbial modules (Park et al., 2019). We used WGCNA analysis to identify modules of co-occurred taxa and relate these modules to traits of musk deer (season, age, and gender). R package WGCNA (version 1.70-3)^6^ was used to perform unsigned WGCNA analysis (Langfelder and Horvath, 2008). The weighted correlation network analysis was performed to cluster OTUs (van Dam et al., 2018).

The soft thresholding power of 8 was chosen based on the criterion of approximate scale-free topology as well as the mean connectivity lower than 100 (Morandin et al., 2016). To divide highly co-occurred OTUs into several module members, hierarchical clustering using the dynamic tree cut method with DeepSplit of 3 was performed to create a hierarchical clustering tree of OTUs as a dendrogram (Do et al., 2017). Module eigengenes were calculated using the moduleEigengenes() function in the WGCNA R package to demonstrate the correlation of the module eigenvalue and OTU abundance profile (Wang et al., 2021). Then, we identified potential modules and OTUs associated with season, age, and gender. The network was generated to visualize the correlations among OTUs in the module associated with season (Liu et al., 2018). The connection weight values (range from 0 to 1) were calculated using the intramodularConnectivity() function in the WGCNA R package to indicate the strength of co-regulation between taxa. Additionally, Cytoscape software (version 3.8.2)^7^ was used for network visualization, with Cytohubba plugin analyzing and extracting the hub OTUs (Kavarthapu et al., 2020).

## Results

### Assessment of Sequence Data

A total of 36,862,468 (140,696 reads/sample) and 12,912,306 (143,470 reads/sample) high-quality clean reads were obtained in FMD and the AMD samples, respectively. The rarefaction curves of sobs and Shannon indexes at the OTU levels became gradually placid as the sequencing depth increased (Supplementary Figure 1). The results demonstrated that each fecal sample had sufficient OTUs to reflect the maximum level of bacterial diversity, which indicated a sufficient sequencing depth.

A total of 3,548 and 2,259 OTUs were identified in FMD and AMD, respectively. At a 97% sequence identity threshold, the OTUs of the FMD were classified into 20 phyla, 35 classes, 93 orders, 171 families, and 404 genera, while those of the AMD were classified into 17 phyla, 27 classes, 66 orders, 125 families, and 300 genera.

### Gut Microbiota Composition in FMD and AMD Across Seasons

**FIGURE 1:**
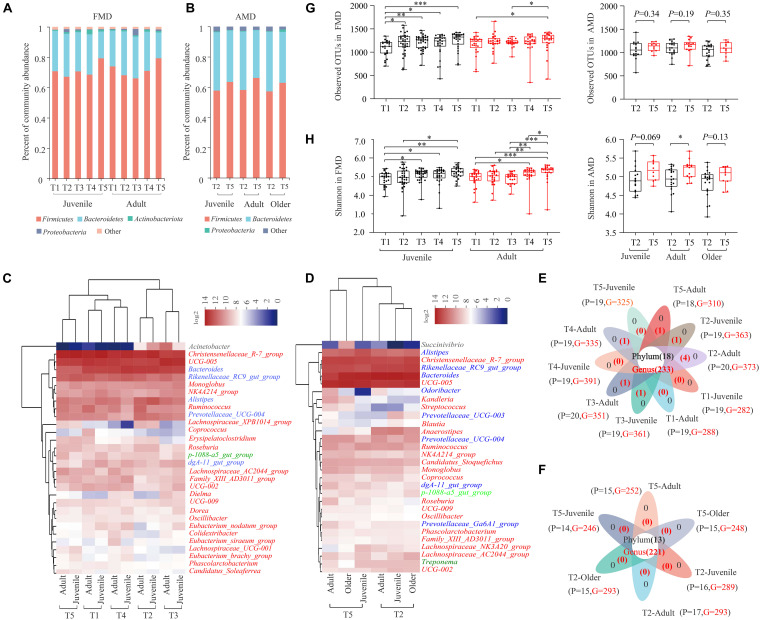
Gut microbial composition of the FMD and AMD. Histogram of relative abundance of individual bacteria phyla of the FMD **(A)** and the AMD **(G)**. Relative abundance of dominant phyla in the FMD **(A)** and the AMD **(B)**. Heat map analysis based on identifiable bacterial genera with the relative abundance of top30 for the FMD **(C)** and the AMD **(D)**. The red, blue, light green, black, and dark green letters represent phylum *Firmicutes*, *Bacteroidetes*, *Planctomycetes*, *Proteobacteria*, and *Spirochaetes*, respectively. Analysis of core and unique bacteria of the FMD **(E)** and AMD **(F)** at phylum and genus levels *via* Venn plots. Seasonal variation of α diversity in the gut microbiota of the musk deer based on Sobs index **(G)** and Shannon index **(H)**. ^∗^*p* < 0.05 (Wilcoxon rank-sum test), ^∗∗^*p* < 0.01, and ^∗∗∗^*p* < 0.001. ns, not significant.

Sequence analysis showed that phylum *Firmicutes* (71.35 ± 12.19%) and *Bacteroidetes* (24.89 ± 12.04%) were significantly dominant in the 262 fecal samples from captive FMD in different seasons and ages (Figure 1A). Besides, Actinobacteriota (1.20%) and Proteobacteria (1.02%) were also dominant (relative abundance >1%). A heat map analysis based on identifiable bacterial genera with the relative abundance of top30 showed that the relative abundance of the genera *Christensenellaceae R7 group* (13.64%), *UCG 005* (10.44%), and *Bacteroides* (8.46%) was higher than 5%. *Rikenellaceae RC9 gut group* (4.25%), *Alistipes* (3.33%), *Ruminococcus* (2.35%), *Prevotellaceae UCG-004* (2.22%), *Monoglobus* (1.74%), and *NK4A214 group* (1.56%) were also dominant (>1%) (Figure 1C). The juvenile and adult individuals were clustered into one type in five different seasons: winter, early spring, and autumn groups were clustered into one type, while summer and late spring groups were clustered into another type. The dominant bacteria genera in FMD belonged to *Firmicutes* or *Bacteroidetes*. The 10 groups of fecal samples from FMD in different seasons and different ages shared 18 bacteria phyla and 233 bacterial genera, with few unique bacteria phyla and genera (Figure 1E).

Similarly, the phylum *Firmicutes* (60.22 ± 10.04%) and *Bacteroidetes* (36.58 ± 10.26%) also significantly dominated in the 90 fecal samples from captive AMD in different seasons and ages (Figure 1B). The relative abundance of the genera *Bacteroides* (14.47%), *UCG 005* (11.75%), *Rikenellaceae RC9 gut group* (8.12%), and *Christensenellaceae R7 group* (8.11%) were higher than 5%. The genera *Alistipes* (4.20%), *Prevotellaceae UCG-004* (1.59%), *Anaerostipes* (1.55%), *Ruminococcus* (1.52%), and *Candidatus Stoquefichus* (1.17%) were also dominant (Figure 1D). The dominant bacteria genera in AMD also belonged to *Firmicutes* or *Bacteroidetes*. Six groups of fecal samples from AMD in two seasons and different ages shared 13 bacteria phyla and 221 bacterial genera, with no unique bacteria phyla or genera (Figure 1F).

### Analysis of Seasonal Differences of Gut Microbiota

#### Seasonal Variation in α Diversity

The Good’s Coverage index of captive FMD and AMD in different seasons was higher than 99%. Both sobs and Shannon indexes were used to reflect the diversity of gut microbiota of musk deer in different seasons.

For captive juvenile FMD, the α diversity of gut microbiota was significantly lower in early spring than in other periods (*p* < 0.05), and it was higher in winter than in other periods (Figures 1G,H). For adult FMD, the α diversity was significantly higher in winter than in other periods (*p* < 0.05) and lower in summer than in other periods. Overall, the α diversity of gut microbiota in captive FMD was higher in winter and autumn than in spring and summer.

For captive adult AMD, the α diversity of gut microbiota was significantly higher in winter than in late spring (Figures 1G,H) (*p* < 0.05). Besides, the α diversity of gut microbiota was higher in winter than, but not significant, for both juvenile and older AMD.

#### Seasonal Variation in β Diversity

**FIGURE 2:**
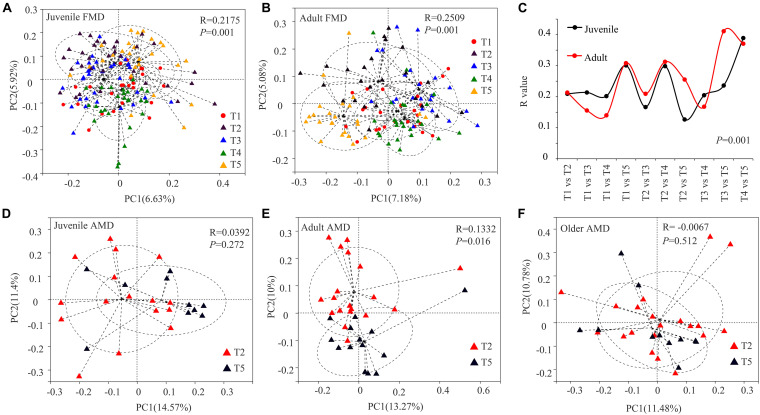
PCoA analysis of gut microbial composition of musk deer in different seasons. Principal coordinate plot between five different periods in juvenile FMD **(A)** and adult FMD **(B)**. **(C)** ANOSIM analysis of gut microbiota between every two seasons in FMD (Supplementary Figures 2, 3). PCoA analysis between late spring and winter groups in juveniles **(D)**, adults **(E)**, and older **(F)** AMD.

The Bray–Curtis distance algorithm was used to calculate the distance between fecal samples of captive musk deer in different seasons. ANOSIM analysis was used to test whether the intergroup differences were significantly greater than the intra-group differences in different seasons. The PCoA analysis showed that the R values were all greater than 0 (*p* = 0.001). Besides, there were significant seasonal differences in the gut microbial composition of the FMD in different seasons, and the intergroup differences were significantly greater than the intra-group differences (Figures 2A–C). The significant differences between the summer and winter groups (*R* = 0.3078, *p* = 0.0010) and the autumn and winter groups (*R* = 0.3254, *p* = 0.001) were relatively high. The gut microbial composition of captive adult AMD was significantly different between the winter and late spring groups (*R* = 0.1332, *p* = 0.016). Moreover, the intergroup difference was significantly greater than the intra-group difference (Figure 2E). However, there was no significant seasonal difference in the composition of gut microbiota in juvenile (*R* = 0.0392, *P* = 0.272) or older (*R* = −0.0067, *P* = 0.512) AMD (Figures 2D,F).

### Analysis of Seasonal Difference of Dominant Bacteria

**FIGURE 3:**
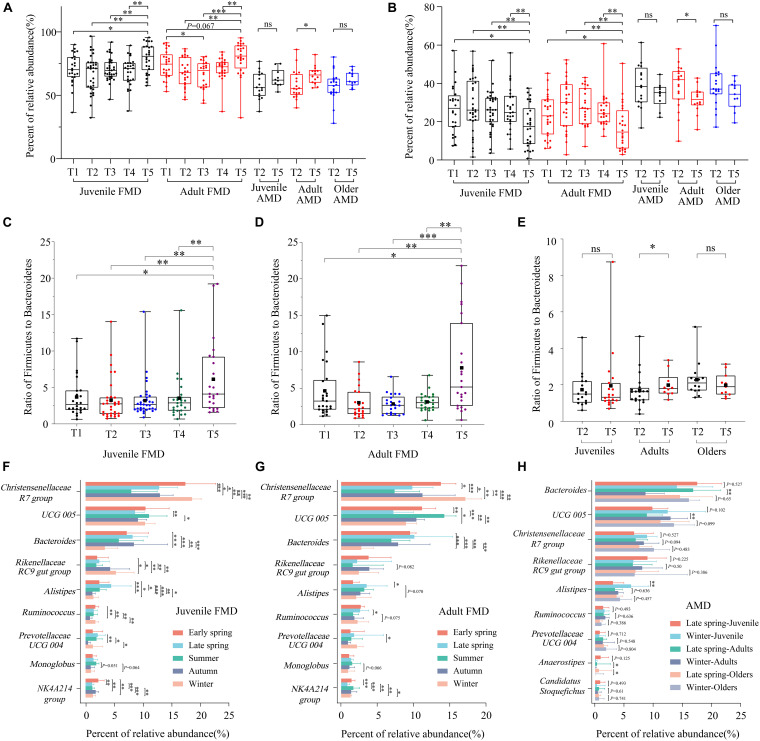
Analysis of seasonal difference of dominant bacteria for captive FMD and AMD. Seasonal difference analysis of *Firmicutes* **(A)** and *Bacteroidetes* **(B)** for FMD and AMD. Seasonal difference analysis of the F/B ratio for juvenile FMD **(C)**, adult FMD **(D)**, and AMD at different ages **(E)** (excluding extreme values). Seasonal difference analysis of dominant bacteria for juvenile FMD **(F)**, adult FMD **(G)**, and AMD at different ages **(H)**. ^∗^*p* < 0.05 (Wilcoxon rank-sum test), ^∗∗^*p* < 0.01, and ^∗∗∗^*p* < 0.001. ns, not significant.

The Wilcoxon rank-sum test was used to analyze the significant seasonal differences. For both juvenile and adult FMD, the relative abundance of *Firmicutes* was significantly higher in winter than in other periods (*p* < 0.05) (Figure 3A), while that of the *Bacteroidetes* showed the opposite result (*p* < 0.05) (Figure 3B). The relative abundance of *Firmicutes* in summer was lower than that in other periods, while that of *Bacteroidetes* showed the opposite result. Overall, the relative abundance of *Firmicutes* in the FMD was higher in autumn, winter, and early spring than in late spring and summer. In contrast, that of *Bacteroidetes* showed the opposite result (Figures 3A,B). Moreover, for captive AMD of different ages, the relative abundance of *Firmicutes* was higher in winter than in late spring, while that of *Bacteroidetes* showed the opposite result. Particularly, there were significant seasonal differences among adult AMD (*p* < 0.05).

The analysis of the ratio of *Firmicutes* to *Bacteroidetes* (F/B ratio) showed that the ratio was significantly higher in winter than in other periods for both juvenile and adult FMD (*p* < 0.05) (Figures 3C,D). Overall, the F/B ratio significantly decreased in summer than in spring and increased in winter than in summer. Similarly, for captive AMD of different ages, the F/B ratio was higher in winter than in late spring, and there were significant seasonal differences among adult AMD (*p* < 0.05) (Figure 3E).

The seasonal difference analysis indicated that nine dominant identifiable bacteria genera in juvenile and adult FMD had significant seasonal differences. The relative abundances of the genera *Christensenellaceae R7 group* and *Ruminococcus* were significantly higher in winter and early spring than in other periods. The relative abundances of the genera *UCG-005* and *Monoglobus* were significantly higher in summer than in other periods. The relative abundance of genus *Alistipes* was significantly higher in late spring and summer than in other periods (Figures 3F,G). Moreover, among the nine dominant bacteria genera in AMD, genera *Bacteroides*, *UCG-005*, *Alistipes*, and *Anaerostipes* showed significant seasonal differences. The relative abundances of genera *UCG-005* and *Alistipes* were significantly higher in winter than in late spring, while those of *Bacteroides* and *Anaerostipes* showed opposite results (Figure 3H).

### Functional Prediction Analysis

The functional prediction analysis in the KEGG database showed that gut microbiota in captive FMD and AMD were mainly involved in carbohydrate metabolism, amino acid metabolism, energy metabolism, metabolism of cofactors and vitamins, metabolism of cofactors and vitamins, translation, and replication and repair. The EggNOG database showed that 17 functions had high relative abundance.

**FIGURE 4:**
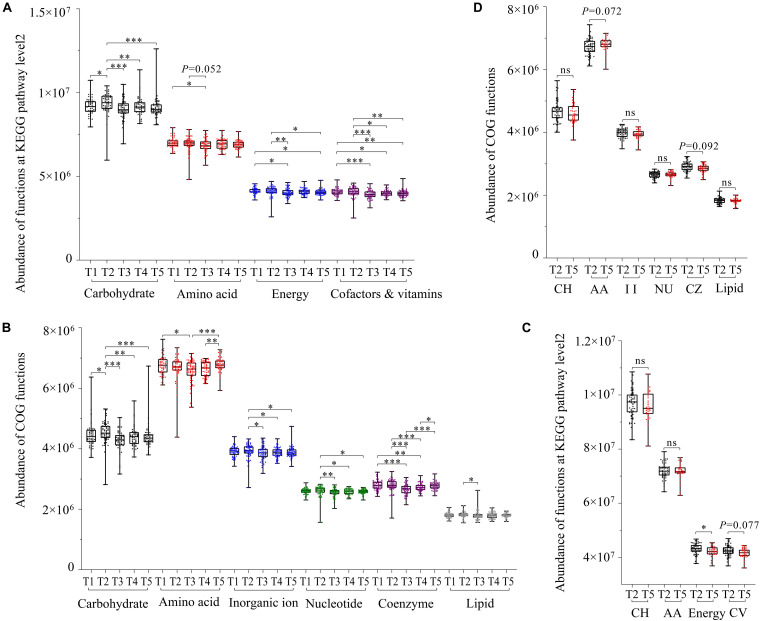
Gut microbial function prediction and seasonal difference analysis based on KEGG and EggNOG databases. Seasonal difference analysis of the main function of gut microbiota of the FMD based on the KEGG database **(A)** and EggNOG database **(B)**. Seasonal difference analysis of the main function of gut microbiota of the AMD based on the EggNOG database **(C)** and KEGG database **(D)**. ^∗^*p* < 0.05 (Wilcoxon rank-sum test), ^∗∗^*p* < 0.01 and ^∗∗∗^*p* < 0.001. ns, not significant.

The KEGG database showed that the four major metabolism-related functions of captive FMD had seasonal differences. Overall, those functions were significantly lower in summer than in other periods (*p* < 0.05) and were relatively higher in spring than in other periods (Figure 4A). The main metabolism-related function of captive AMD was higher in late spring than in winter. Also, the energy metabolic function had significant seasonal differences (*p* < 0.05) (Figure 4C).

The EggNOG database showed that six major metabolism-related functions also had seasonal differences. Overall, the functions were relatively higher in spring than in other periods. All the major metabolic functions of gut microflora in captive FMD were significantly lower in summer than in other periods except for lipid transfer and metabolic function (*p* < 0.05) (Figure 4B).

Similarly, all the major metabolic functions in captive AMD were higher in late spring than in winter except for amino acid transfer and metabolic function (Figure 4D).

### The Hub OTUs Fluctuated With the Season Using WGCNA Analysis

Baseline characteristics of participants in weighted correlation network analysis. A total of 262 FMD and 3493 OTUs were included in the study, with the mean age and the proportion of males of about 2.45 and 75.19%, respectively. The individual in early spring (T1), late spring (T2), summer (T3), autumn (T4), and winter (T5) periods accounted for 18.70, 21.76, 21.37, 19.08, and 19.08%, respectively. The sample dendrogram and trait heat map is shown in Supplementary Figure 4.

The network was constructed using one-step network construction. The networkType was set to signed and soft-threshold power 8 to define the adjacency matrix based on the criterion of approximate scale-free topology (Supplementary Figure 5), with minimum module size 30, the module detection sensitivity DeepSplit set to 3, and cut height for merging of modules of 0.25 which means that the modules whose eigengenes are correlated above 0.75 will be merged (Supplementary Figure 6).

A total of 3,493 OTUs were parsed into 13 different color modules. Among these 13 modules, the gray module indicated unassigned bacterial taxa. In the dendrogram, each leaf, represented as a short vertical line, corresponded to a bacterial taxon. Densely interconnected branches of the dendrogram group represented highly co-occurring bacterial taxa (Supplementary Figure 7).

**FIGURE 5:**
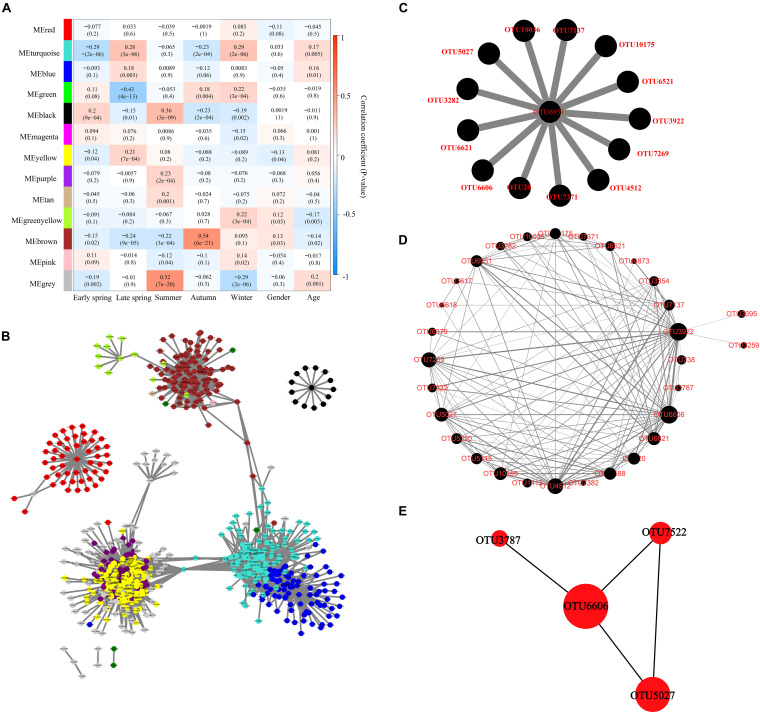
Identification of key module and hub OTUs based on WGCNA. **(A)** Correlation between module eigenvalue and traits of musk deer. Depth of color corresponds to depth of correlation and *p*-value of each module presented in parentheses. **(B)** Network diagram of the hub OTUs. Each node represented the OTUs whose Ktotal value was in the top 10%, and its color represented the corresponding module. The gray line thickness represents the weight value between nodes (OTUs). **(C)** Visualization of hub OTUs in black modules. **(D)** Visualization of full weighted networks in black modules associated with different seasons. The node size represented the Kwithin value of the node in the black module, that is, the size of the connectivity in the module. The gray line thickness represented the weight value between nodes (OTUs). **(E)** Identification of key OTU in the black module through Cytohubba. The node size represented the Kwithin value, and the shade of red indicated the importance within the module.

The correlation between module eigenvalue and trait was calculated. The module–trait relationship heat map demonstrated the correlation coefficient between module eigenvalues and traits (−1 to 1). The black module was significantly correlated with five different periods simultaneously (*p* < 0.01) and was not correlated with other traits (age and gender) (Figure 5A). Therefore, the black module was selected for subsequent analysis.

Furthermore, the OTU in the top 10% of global importance [Ktotal (the global network connectivity) value accounted for the top 10%] in WGCNA was extracted, and the network diagram was constructed in Cytoscape (715 nodes and 9,447 edges). The results showed that the black module was relatively independent and had low correlation with other modules (Figure 5B). The black module had the highest correlation with seasonal change, and 14 OTUs in the global hub OTUs were located in the black module, which was significantly related to seasonal changes (Figure 5C). OTUs with a connectivity weight value greater than 0.02 in black modules were extracted to construct the network diagram of black modules (Figure 5D). The thickness of gray lines represented the co-occurred strength between OTUs. The size of an OTU node represented the Kwithin (the intramodular connectivity) value and the connectivity between the node and the black module. CytoHubba plugin was further used to extract and identify the hub OTUs including OTU6606, OTU5027, OTU7522, and OTU3787 (Figure 5E) as top 4, which belong to the order *Oscillospirales* in phylum *Firmicutes*, and the first three hub OTUs belong to genus *UCG-005* of family *Oscillospiraceae*.

## Discussion

The normal steady-state gut microbiota is closely related to the health of the host and coevolves in a mutualistic relationship with the host through complex interactions. Its unique community structure and metabolites are essential for regulating host metabolism, growth, and development, resistance to pathogens, immune regulation, adaptation, evolution, etc. (Bäckhed et al., 2005; Engel et al., 2012; Ezenwa et al., 2012; Zhang et al., 2016). This study systematically and comprehensively analyzed the seasonal differences of gut microbiota in captive FMD and AMD at different ages. The results indicated that the FMD and AMD shared seven dominant bacteria genera, including the genera *Bacteroides*, *Christensenellaceae R7 group*, *UCG 005*, *Rikenellaceae RC9 gut group*, *Alistipes*, *Ruminococcus*, and *Prevotellaceae UCG 004* and genera *Christensenellaceae R7 group*, *Ruminococcus*, and *Prevotellaceae UCG 004*. Current studies have shown that genus *Christensenellaceae R7 group* is widely found in the intestinal tract and mucosa of the host, which is essential for good health and is involved in amino acid and lipid metabolisms (Waters and Ley, 2019). Genus *Ruminococcus* is involved in the degradation of cellulose and hemicellulose in the rumen of ruminants (Matulova et al., 2008; La Reau et al., 2016). It produces several cellulases and hemicellulases that convert dietary fiber into various nutrients (La Reau and Suen, 2018). It is also involved in food digestion and carbohydrate metabolism in ruminants. Genus *Prevotellaceae UCG 004* is involved in the degradation of polysaccharides and the production of short-chain fatty acids (SCFAs) (Heinritz et al., 2016). Family *Christensenellaceae* (genus *Christensenellaceae R7 group* belongs to this family), *Ruminococcaceae* (*Ruminococcus* belongs to this family), and genus *Alistipes* are associated with immune regulation and healthy homeostasis and are regarded as potentially beneficial bacteria (Kong et al., 2016; Shang et al., 2016; Wang et al., 2018). Among them, the genera *Christensenellaceae R7 group* and *Alistipes* are classified as potential biomarkers for intestinal diseases (Crohn’s disease, colorectal cancer, Clostridium difficile infection, etc.) (Mancabelli et al., 2017). Moreover, the genera *Bacteroides*, *Alistipes*, and *Rikenellaceae RC9 gut group* belong to *Bacteroidetes*. Genus *Bacteroides* can improve the metabolism of the host, mainly by participating in the metabolism of bile acid, protein, and fat, and regulating carbohydrate metabolism. Genus *Alistipes* is involved in the metabolism of SCFAs. *Bacteroides* and *Alistipes* belong to bile-tolerant microorganisms (David et al., 2014). High-fat animal feed can increase the relative abundance of genera *Bacteroides* and *Alistipes* (Wan et al., 2019), thus improving lipid metabolism by regulating acetic acid production (Yin et al., 2018). Genus *Rikenellaceae RC9 gut group* also promotes lipid metabolism (Zhou et al., 2018).

Furthermore, phyla *Firmicutes* and *Bacteroidetes* significantly dominated the 352 fecal samples from captive musk deer in different seasons (relative abundance of more than 90%), consistent with other studies (Hu et al., 2017; Zhao et al., 2019; Fountain-Jones et al., 2020). *Firmicutes* and *Bacteroidetes* are the dominant core bacteria in the rumen of ruminants, with the highest relative abundance. They are involved in essential processes, such as food digestion, nutrient regulation, and absorption, energy metabolism, and defense against invasion of foreign pathogens in the gastrointestinal tract of hosts (Jewell et al., 2015; Guan et al., 2017; Wang et al., 2017). *Firmicutes* promote fiber degradation in food and convert cellulose into volatile fatty acids, enhancing food digestion and growth and development. Herein, among the top 30 bacterial genera of gut microbiota, 23 and 19 were *Firmicutes* in the FMD and AMD, respectively. Besides, most were associated with carbohydrate metabolism and cellulose digestion and absorption. *Bacteroidetes* mainly promote the digestion and absorption of proteins and carbohydrates in the food, thus promoting the development of the gastrointestinal immune system. Among the top 30 identifiable bacterial genera, five and eight were *Bacteroidetes* in the FMD and AMD, respectively, of which most were involved in lipid metabolism. The nutrient level of animal feed directly affects the abundance of *Firmicutes* and *Bacteroidetes*. Herein, *Firmicutes* and *Bacteroides* were the core dominant microflora in the FMD and AMD at different seasons and ages. In addition, the relative abundances of *Firmicutes* and *Bacteroidetes* and their ratios had significant seasonal differences. The relative abundances of *Firmicutes* and the F/B ratio were higher in the cold season than in the warm season. This is beneficial for captive FMD and AMD to adapt to the cold season, thus promoting the decomposition of cellulose and hemicellulose in feed, carbohydrate metabolism, and nutrient digestion and absorption.

The FMD and AMD are typical ruminants. Wild individuals feed on various high-fiber leaves, while captive individuals feed on concentrated animal feed and rouge feed. The former mainly includes carrot (*Daucus carota*), potato (*Solanum tuberosum*), maize (*Zea mays*), soybean (*Glycine max*), etc., and the latter includes fresh leaves (warm season) or dry leaves (cold season). Core and dominant bacteria genera are essential in food digestion, nutrient absorption, and energy metabolism of captive FMD and AMD. Herein, the relative abundances of the genera *Christensenellaceae R7 group*, *Ruminococcus*, and *Prevotellaceae UCG 004* (*Firmicutes*) were higher in the cold season than in the warm season. In contrast, the relative abundance of genus *Bacteroides* (*Bacteroidetes*) showed the opposite results, indicating that the seasonal difference of dominant bacteria genera is closely associated with animal feed composition in different periods.

Principal coordinate analysis showed that both adult and juvenile musk deer had significant seasonal differences, indicating that the gut microbial structure and composition of FMD and AMD are significantly different at different seasons, consistent with other studies (Amato et al., 2015; Maurice et al., 2015; Trosvik et al., 2018). The seasonal difference is closely related to food resources, dietary structure, nutrient utilization, and feeding pattern (Xue et al., 2015; Wu et al., 2017; Kartzinel et al., 2019). The Wilcoxon rank-sum test showed that the α diversity of the gut microbiota had significant seasonal differences in captive FMD and AMD. Overall, the α diversity of the gut microbiota in musk deer was higher in winter and autumn than in spring and summer. Previous studies have shown that higher α diversity leads to a more complex and stable intestinal microbiota composition, enhancing resistance to external interference, and adaptability, which is beneficial to the host health (Stoffel et al., 2020). The decrease or loss of α diversity is associated with various diseases (Rogers et al., 2016). Therefore, the reported higher α-diversity of the gut microbiota in captive FMD and AMD in autumn and winter can improve the resistance to adverse environmental factors, reduce the influence of adverse environmental factors, and promote the intake of fiber-rich food and nutrient absorption and utilization in the cold season.

The function prediction analysis based on KEGG and EggNOG databases showed that the gut microorganisms in captive FMD and AMD mainly involve carbohydrate metabolism, amino acid metabolism, energy metabolism, and cofactor and vitamin metabolism. These metabolic functions also had seasonal differences and were significantly correlated with core bacteria phyla and genera. The seasonal changes of core bacteria were also closely associated with the seasonal function changes. The effect of season variation on gut microbiota of musk deer was higher than that of age and gender based on the correlation between module eigenvalue and traits of musk deer. WGCNA analysis indicated that the black module had low correlation with other modules and has the highest correlation with seasons. OTU6606, OTU5027, OTU7522, and OTU3787 were significantly fluctuated with the season, which all belong to phyla *Firmicutes*. The four hub OTUs were at the core of the black module and can greatly affect the network structure of the co-occurrence bacterial taxa network in the black module, which can be used as an important indicator to evaluate the intestinal health of captive forest musk deer.

## Conclusion

The study systematically and comprehensively analyzed the seasonal differences of gut microbiota structure and function through 16S rRNA gene sequencing of 352 fecal samples from the captive FMD and AMD at different ages. Comparison analysis identified significant seasonal variations of α-diversity, core microbiota, and metabolic functions. The α-diversity, phylum *Firmicutes*, and F/B ratio in the gut microbiota of both musk deer were higher in the cold season than in the warm season. The major metabolism-related functions were also significantly higher in the cold season than in the warm season in FMD. The four identified hub OTUs can be used as an important indicator to evaluate the intestinal health of captive forest musk deer. Therefore, this study provides insights into the captive breeding environment and future reintroduction programs. Besides, functional annotation analysis of gut microbiota and the seasonal changes of metabolic pathways combined with metagenome and metabolomic analyses can help to further explore the role of gut microbiota in the health, environmental adaptation, and metabolism of the FMD and AMD in the future.

## Data Availability Statement

The data presented in the study are deposited in the NCBI GenBank, accession number PRJNA725631 (https://dataview.ncbi.nlm.nih.gov/object/PRJNA725631?review er=e2rcdic27nir1a7qhp8unlk9s6).

## Ethics Statement

A total of 262 fresh feces of captive forest musk deer were collected with the permission of the authorities of the forest musk deer breeding center in Qilian county, China. Also, a total of 90 fecal samples of captive alpine musk deer were collected with the permission of the authorities of the alpine musk deer breeding center in Yuzhong county, China. All procedures were approved by the Ethical Committee for Experimental Animal Welfare of the Northwest Institute of Plateau Biology.

## Author Contributions

FJ and TZ conceived and designed the experiments. FJ wrote the first draft of the manuscript. FJ, HG, WQ, DL, JZ, and DW contributed to the sampling and laboratory work. FJ, PS, and HW conducted the data analysis. All authors have read and approved the final manuscript.

## Conflict of Interest

The authors declare that the research was conducted in the absence of any commercial or financial relationships that could be construed as a potential conflict of interest.

## Publisher’s Note

All claims expressed in this article are solely those of the authors and do not necessarily represent those of their affiliated organizations, or those of the publisher, the editors and the reviewers. Any product that may be evaluated in this article, or claim that may be made by its manufacturer, is not guaranteed or endorsed by the publisher.

## Footnotes

1
http://drive5.com/uparse/

2
https://www.arb-silva.de/fileadmin/silva_databases/release_138/Exports/SILVA_138_SSURef_NR99_tax_silva.fasta.gz

3
https://forum.qiime2.org/t/processing-filtering-and-evaluating-the-silva-database-and-other-reference-sequence-data-with-rescript/15494

4
http://rdp.cme.msu.edu/

5
http://qiime.org/scripts/assign_taxonomy

6
https://cran.r-project.org/web/packages/WGCNA/

7
http://www.cytoscape.org/

## Supplementary Material

Supplementary Figure 1 Rarefaction curves based on Sobs (the observed richness) index and Shannon index at the OTU level of forest musk deer **(A,B)** and alpine musk deer **(C,D)**.

Supplementary Figure 2 PCoA analysis of gut microbial composition between every two seasons in juvenile FMD.

Supplementary Figure 3 PCoA analysis of gut microbial composition between every two seasons in adult FMD.

Supplementary Figure 4 Sample dendrogram and trait heatmap.

Supplementary Figure 5 Analysis of network topology for various soft-thresholding powers.

Supplementary Figure 6 Clustering dendrogram of bacterial taxa, with dissimilarity based on topological overlap, together with assigned module colors.

Supplementary Figure 7 Module membership identification using dynamic tree.

## References

[B1] Amato K. R. Leigh S. R. Kent A. Mackie R. I. Yeoman C. J. Stumpf R. M. (2015). The gut microbiota appears to compensate for seasonal diet variation in the wild black howler monkey (*Alouatta pigra*). *Microb. Ecol.* 69 434–443. 10.1007/s00248-014-0554-7 25524570

[B2] Bäckhed F. Ley R. E. Sonnenburg J. L. Peterson D. A. Gordon J. I. (2005). Host-bacterial mutualism in the human intestine. *Science* 307 1915–1920. 10.1126/science.1104816 15790844

[B3] Bolger A. M. Lohse M. Usadel B. (2014). Trimmomatic: a flexible trimmer for illumina sequence data. *Bioinformatics* 30 2114–2120. 10.1093/bioinformatics/btu170 24695404 PMC4103590

[B4] Cai Y. H. Yang J. D. Wang J. M. Yang Y. Fu W. L. Zheng C. L. (2020). Changes in the population genetic structure of captive forest musk deer (*Moschus berezovskii*) with the increasing number of generation under closed breeding conditions. *Animals* 10:2. 10.3390/ani10020255 32033449 PMC7071047

[B5] Caporaso J. G. Kuczynski J. Stombaugh J. Bittinger K. Bushman F. D. Costello E. K. (2010). QIIME allows analysis of high-throughput community sequencing data. *Nat. Methods* 7 335–336. 10.1038/nmeth.f.303 20383131 PMC3156573

[B6] Claesson M. J. Jeffery I. B. Conde S. Power S. E. O’Connor E. M. Cusack S. (2012). Gut microbiota composition correlates with diet and health in the elderly. *Nature* 488:178. 10.1038/nature11319 22797518

[B7] Costello E. K. Lauber C. L. Hamady M. Fierer N. Gordon J. I. Knight R. (2009). Bacterial community variation in human body habitats across space and time. *Science* 326 1694–1697. 10.1126/science.1177486 19892944 PMC3602444

[B8] David L. A. Maurice C. F. Carmody R. N. Gootenberg D. B. Button J. E. Wolfe B. E. (2014). Diet rapidly and reproducibly alters the human gut microbiome. *Nature* 505 559–563. 10.1038/nature12820 24336217 PMC3957428

[B9] Do D. N. Dudemaine P. L. Li R. Ibeagha-Awemu E. M. (2017). Co-expression network and pathway analyses reveal important modules of miRNAs regulating milk yield and component traits. *Int. J. Mol. Sci.* 18:1560. 10.3390/ijms18071560 28718798 PMC5536048

[B10] Engel P. Martinson V. G. Moran N. A. (2012). Functional diversity within the simple gut microbiota of the honey bee. *Proc. Natl. Acad. Sci. U. S. A.* 109 11002–11007. 10.1073/pnas.1202970109 22711827 PMC3390884

[B11] Ezenwa V. O. Gerardo N. M. Inouye D. W. Medina M. Xavier J. B. (2012). Animal behavior and the microbiome. *Science* 338 198–199. 10.1126/science.1227412 23066064

[B12] Fan J. M. Zheng X. L. Wang H. Y. Qi H. Jiang B. M. Qiao M. P. (2019). Analysis of genetic diversity and population structure in three forest musk deer captive populations with different origins. *G3 (Bethesda)* 9 1037–1044. 10.1534/g3.119.400001 30737238 PMC6469423

[B13] Fan Z. X. Li W. J. Jin J. Z. Cui K. Yan C. C. Peng C. J. (2018). The draft genome sequence of forest musk deer (Moschus berezovskii). *Gigascience* 7 1–6. 10.1093/gigascience/giy038 29635287 PMC5906906

[B14] Fountain-Jones N. M. Clark N. J. Kinsley A. C. Carstensen M. Forrester J. Johnson T. J. (2020). Microbial associations and spatial proximity predict North American moose (*Alces alces*) gastrointestinal community composition. *J. Anim. Ecol.* 89 817–828. 10.1111/1365-2656.13154 31782152

[B15] Guan Y. Yang H. T. Han S. Y. Feng L. M. Wang T. M. Ge J. P. (2017). Comparison of the gut microbiota composition between wild and captive sika deer (*Cervus nippon hortulorum*) from feces by high-throughput sequencing. *AMB Express* 7:212. 10.1186/s13568-017-0517-8 29170893 PMC5700909

[B16] Guo W. Mishra S. Wang C. D. Zhang H. M. Ning R. H. Kong F. L. (2019). Comparative study of gut microbiota in wild and captive giant pandas (*Ailuropoda melanoleuca*). *Genes* 10:827. 10.3390/genes10100827 31635158 PMC6826394

[B17] Harris R. (2016). *Moschus chrysogaster. The IUCN Red List of Threatened Species 2016: e.T13895A61977139*. 10.2305/IUCN.UK.2016-1.RLTS.T13895A61977139.en (accessed February 20, 2021). 32055894

[B18] Heinritz S. N. Weiss E. Eklund M. Aumiller T. Louis S. Rings A. (2016). Intestinal microbiota and microbial metabolites are changed in a pig model fed a high-fat/low-fiber or a low-fat/high-fiber diet. *PLoS One* 11:0154329. 10.1371/journal.pone.0154329 27100182 PMC4839692

[B19] Hu X. L. Liu G. Shafer A. B. A. Wei Y. T. Zhou J. T. Lin S. B. (2017). Comparative analysis of the gut microbial communities in forest and alpine musk deer using high-throughput sequencing. *Front. Microbiol.* 8:572. 10.3389/fmicb.2017.00572 28421061 PMC5376572

[B20] Jewell K. A. McCormick C. A. Odt C. L. Weimer P. J. Suen G. (2015). Ruminal bacterial community composition in dairy cows is dynamic over the course of two lactations and correlates with feed efficiency. *Appl. Environ. Microb.* 81 4697–4710. 10.1128/AEM.00720-15 25934629 PMC4551193

[B21] Jiang F. Zhang J. J. Gao H. M. Cai Z. Y. Zhou X. W. Li S. Q. (2020). Musk deer (*Moschus* spp.) face redistribution to higher elevations and latitudes under climate change in China. *Sci. Total. Environ.* 704:135335. 10.1016/j.scitotenv.2019.135335 31784177

[B22] Jiang Z. G. Jiang J. P. Wang Y. Z. Zhang E. Zhang Y. Y. Li L. L. (2016). Red list of China’s vertebrates. *Biodivers. Sci.* 24 500–551. 10.17520/biods.2016076 (in Chinese), 34063014

[B23] Kartzinel T. R. Hsing J. C. Musili P. M. Brown B. R. P. Pringle R. M. (2019). Covariation of diet and gut microbiome in African megafauna. *Proc. Natl. Acad. Sci. U. S. A.* 116 23588–23593. 10.1073/pnas.1905666116 31685619 PMC6876249

[B24] Kavarthapu R. Anbazhagan R. Sharma A. K. Shiloach J. Dufau M. L. (2020). Linking phospho-gonadotropin regulated testicular rna helicase (GRTH/DDX25) to histone ubiquitination and acetylation essential for spermatid development during spermiogenesis. *Front. Cell Dev. Biol.* 8:310. 10.3389/fcell.2020.00310 32478068 PMC7242631

[B25] Kong F. Hua Y. Zeng B. Ning R. Li Y. Zhao J. (2016). Gut microbiota signatures of longevity. *Curr. Biol.* 26 R832–R833. 10.1016/j.cub.2016.08.015 27676296

[B26] La Reau A. J. Meier-Kolthoff J. P. Suen G. (2016). Sequence-based analysis of the genus Ruminococcus resolves its phylogeny and reveals strong host association. *Microb. Genom.* 2:e000099. 10.1099/mgen.0.000099 28348838 PMC5359413

[B27] La Reau A. J. Suen G. (2018). The ruminococci: key symbionts of the gut ecosystem. *J. Microbiol.* 56 199–208. 10.1007/s12275-018-8024-4 29492877

[B28] Langfelder P. Horvath S. (2008). WGCNA: an R package for weighted correlation network analysis. *BMC Bioinformatics* 9:559. 10.1186/1471-2105-9-559 19114008 PMC2631488

[B29] Langille M. G. I. Zaneveld J. Caporaso J. G. McDonald D. Knights D. Reyes J. A. (2013). Predictive functional profiling of microbial communities using 16S rRNA marker gene sequences. *Nat. Biotechnol.* 31 814–821. 10.1038/nbt.2676 23975157 PMC3819121

[B30] Li Y. M. Hu X. L. Yang S. Zhou J. T. Zhang T. X. Qi L. (2017). Comparative analysis of the gut microbiota composition between captive and wild forest musk deer. *Front. Microbiol.* 8:1705. 10.3389/fmicb.2017.01705 28928728 PMC5591822

[B31] Liu T. Zhang A. N. Wang J. W. Liu S. F. Jiang X. T. Dang C. Y. (2018). Integrated biogeography of planktonic and sedimentary bacterial communities in the Yangtze River. *Microbiome* 6:16. 10.1186/s40168-017-0388-x 29351813 PMC5775685

[B32] Magoč T. Salzberg S. L. (2011). Flash: fast length adjustment of short reads to improve genome assemblies. *Bioinformatics* 27 2957–2963. 10.1093/bioinformatics/btr507 21903629 PMC3198573

[B33] Mancabelli L. Milani C. Lugli G. A. Turroni F. Cocconi D. van Sinderen D. (2017). Identification of universal gut microbial biomarkers of common human intestinal diseases by meta-analysis. *FEMS Microbiol. Ecol.* 93:fix153. 10.1093/femsec/fix153 29126267

[B34] Matulova M. Nouaille R. Capek P. Pean M. Delort A. M. Forano E. (2008). NMR study of cellulose and wheat straw degradation by Ruminococcus albus 20. *FEBS J.* 275 3503–3511. 10.1111/j.1742-4658.2008.06497.x 18513327

[B35] Maurice C. F. Knowles S. C. L. Ladau J. Pollard K. S. Fenton A. Pedersen A. B. (2015). Marked seasonal variation in the wild mouse gut microbiota. *ISME J.* 9 2423–2434. 10.1038/ismej.2015.53 26023870 PMC4611506

[B36] Morandin C. Tin M. M. Y. Abril S. Gomez C. Pontieri L. Schiott M. (2016). Comparative transcriptomics reveals the conserved building blocks involved in parallel evolution of diverse phenotypic traits in ants. *Genome Biol.* 17:43. 10.1186/s13059-016-0902-7 26951146 PMC4780134

[B37] Nicholson J. K. Holmes E. Kinross J. Burcelin R. Gibson G. Jia W. (2012). Host-gut microbiota metabolic interactions. *Science* 336 1262–1267. 10.1126/science.1223813 22674330

[B38] Oksanen J. Blanchet F. G. Friendly M. Kindt R. Legendre P. Mcglinn D. (2019). *vegan: Community ecology package, R package Version 2.5-6*. Available online at: https://CRAN.R-project.org/package=vegan

[B39] O’Toole P. W. Jeffery I. B. (2015). Gut microbiota and aging. *Science* 350 1214–1215. 10.1126/science.aac8469 26785481

[B40] Park C. H. Lee J. G. Lee A. R. Eun C. S. Han D. S. (2019). Network construction of gastric microbiome and organization of microbial modules associated with gastric carcinogenesis. *Sci. Rep.* 9:12444. 10.1038/s41598-019-48925-4 31455798 PMC6712011

[B41] Perry M. (2016). *heatmaps: Flexible heatmaps for functional genomics and sequence features, R Package Version 1.11.0*. Available online at: https://rdrr.io/bioc/heatmaps/

[B42] Rogers M. B. Firek B. Shi M. Yeh A. Brower-Sinning R. Aveson V. (2016). Disruption of the microbiota across multiple body sites in critically ill children. *Microbiome* 4:66. 10.1186/s40168-016-0211-0 28034303 PMC5200963

[B43] Saris C. G. J. Horvath S. van Vught P. W. J. van Es M. A. Blauw H. M. Fuller T. F. (2009). Weighted gene co-expression network analysis of the peripheral blood from amyotrophic lateral sclerosis patients. *BMC Genomics* 10:405. 10.1186/1471-2164-10-405 19712483 PMC2743717

[B44] Shang Q. Shan X. Cai C. Hao J. Li G. Yu G. (2016). Dietary fucoidan modulates the gut microbiota in mice by increasing the abundance of lactobacillus and ruminococcaceae. *Food Funct.* 7 3224–3232. 10.1039/C6FO00309e 27334000

[B45] Stoffel M. A. Acevedo-Whitehouse K. Morales-Duran N. Grosser S. Chakarov N. Krueger O. (2020). Early sexual dimorphism in the developing gut microbiome of northern elephant seals. *Mol. Ecol.* 29 2109–2122. 10.1111/mec.15385 32060961

[B46] Sun X. N. Cai R. B. Jin X. L. Shafer A. B. A. Hu X. L. Yang S. (2018). Blood transcriptomics of captive forest musk deer (*Moschus berezovskii*) and possible associations with the immune response to abscesses. *Sci. Rep.* 8:599. 10.1038/s41598-017-18534-0 29330436 PMC5766596

[B47] Sun Y. W. Sun Y. J. Shi Z. H. Liu Z. S. Zhao C. Lu T. F. (2020). Gut microbiota of wild and captive alpine musk deer (*Moschus chrysogaster*). *Front. Microbiol.* 10:3156. 10.3389/fmicb.2019.03156 32038587 PMC6985557

[B48] Trosvik P. de Muinck E. J. Rueness E. K. Fashing P. J. Beierschmitt E. C. Callingham K. R. (2018). Multilevel social structure and diet shape the gut microbiota of the gelada monkey, the only grazing primate. *Microbiome* 6:84. 10.1186/s40168-018-0468-6 29729671 PMC5935910

[B49] van Dam S. Vosa U. van der Graaf A. Franke L. de Magalhaes J. P. (2018). Gene co-expression analysis for functional classification and gene-disease predictions. *Brief Bioinform.* 19 575–592. 10.1093/bib/bbw139 28077403 PMC6054162

[B50] Wan Y. Wang F. L. Yuan J. H. Li J. Jiang D. D. Zhang J. J. (2019). Effects of dietary fat on gut microbiota and faecal metabolites, and their relationship with cardiometabolic risk factors: a 6-month randomised controlled-feeding trial. *Gut* 68 1417–1429. 10.1136/gutjnl-2018-317609 30782617

[B51] Wang H. J. Liu D. X. Song P. F. Jiang F. Chi X. W. Zhang T. Z. (2021). Exposure to hypoxia causes stress erythropoiesis and downregulates immune response genes in spleen of mice. *BMC Genomics* 22:413. 10.1186/s12864-021-07731-x 34090336 PMC8178839

[B52] Wang X. F. Hao Y. L. Liu X. X. Yu S. J. Zhang W. B. Yang S. T. (2019). Gut microbiota from end-stage renal disease patients disrupt gut barrier function by excessive production of phenol. *J. Genet. Genomics* 46 409–412. 10.1016/j.jgg.2019.03.015 31466928

[B53] Wang Y. Harris R. (2015). *Moschus berezovskii. The IUCN Red List of Threatened Species 2015: e.T13894A103431781.* 10.2305/IUCN.UK.2015-4.RLTS.T13894A61976926.en (accessed February 20, 2021). 32055894

[B54] Wang Y. J. Zhang H. Zhu L. Xu Y. L. Liu N. Sun X. M. (2018). Dynamic distribution of gut microbiota in goats at different ages and health states. *Front. Microbiol.* 9:2509. 10.3389/fmicb.2018.02509 30405569 PMC6207909

[B55] Wang Y. Y. Cao P. H. Wang L. Zhao Z. Y. Chen Y. L. Yang Y. X. (2017). Bacterial community diversity associated with different levels of dietary nutrition in the rumen of sheep. *Appl. Microbiol. Biot.* 101 3717–3728. 10.1007/s00253-017-8144-5 28175950

[B56] Waters J. L. Ley R. E. (2019). The human gut bacteria Christensenellaceae are widespread, heritable, and associated with health. *BMC Biol.* 17:83. 10.1186/s12915-019-0699-4 31660948 PMC6819567

[B57] Wu J. Y. Wang W. (2006). *The Musk Deer of China.* Beijing: China forestry publishing house.

[B58] Wu Q. Wang X. Ding Y. Hu Y. B. Nie Y. G. Wei W. (2017). Seasonal variation in nutrient utilization shapes gut microbiome structure and function in wild giant pandas. *Proc. Biol. Sci.* 284:20170955. 10.1098/rspb.2017.0955 28904137 PMC5597826

[B59] Xue Z. S. Zhang W. P. Wang L. H. Hou R. Zhang M. H. Fei L. S. (2015). The bamboo-eating giant panda harbors a carnivore-like gut microbiota, with excessive seasonal variations. *mBio* 6 e00022–15. 10.1128/mBio.00022-15 25991678 PMC4442137

[B60] Yang Q. S. Meng X. X. Xia L. Feng Z. J. (2003). Conservation status and causes of decline of musk deer (*Moschus* spp.) in China. *Biol. Conserv.* 109 333–342. 10.1016/S0006-3207(02)00159-3

[B61] Yin J. Li Y. Y. Han H. Chen S. Gao J. Liu G. (2018). Melatonin reprogramming of gut microbiota improves lipid dysmetabolism in high-fat diet-fed mice. *J. Pineal. Res.* 65:e12524. 10.1111/jpi.12524 30230594

[B62] Yoo J. Y. Groer M. Dutra S. V. O. Sarkar A. McSkimming D. I. (2020). Gut microbiota and immune system interactions. *Microorganisms* 8:1587. 10.3390/microorganisms8101587 33076307 PMC7602490

[B63] Zhang C. H. Zhang M. H. Wang S. Y. Han R. J. Cao Y. F. Hua W. Y. (2010). Interactions between gut microbiota, host genetics and diet relevant to development of metabolic syndromes in mice. *ISME J.* 4 232–241. 10.1038/ismej.2009.112 19865183

[B64] Zhang L. Jie H. Xiao Y. P. Zhou C. Q. Lyu W. T. Bai W. K. (2019). Genomic identification and expression analysis of the cathelicidin gene family of the forest musk deer. *Animals* 9:481. 10.3390/ani9080481 31344924 PMC6719980

[B65] Zhang Z. G. Xu D. M. Wang L. Hao J. J. Wang J. F. Zhou X. (2016). Convergent evolution of rumen microbiomes in high-altitude mammals. *Curr. Biol.* 26 1873–1879. 10.1016/j.cub.2016.05.012 27321997

[B66] Zhao G. J. Ma T. Y. Tang W. J. Li D. Y. Mishra S. K. Xu Z. X. (2019). Gut microbiome of chinese forest musk deer examined across gender and age. *Biomed. Res. Int.* 2019:9291216. 10.1155/2019/9291216 31886268 PMC6925676

[B67] Zhao K. L. Liu Y. Zhang X. Y. Palahati P. Wang H. N. Yue B. S. (2011). Detection and characterization of antibiotic-resistance genes in Arcanobacterium pyogenes strains from abscesses of forest musk deer. *J. Med. Microbiol.* 60 1820–1826. 10.1099/jmm.0.033332-0 21852523

[B68] Zhou L. Y. Xiao X. H. Zhang Q. Zheng J. Li M. Yu M. (2018). Improved glucose and lipid metabolism in the early life of female offspring by maternal dietary genistein is associated with alterations in the gut microbiota. *Front. Endocrinol.* 9:516. 10.3389/fendo.2018.00516 30233500 PMC6131301

[B69] Zmora N. Suez J. Elinav E. (2019). You are what you eat: diet, health and the gut microbiota. *Nat. Rev. Gastro. Hepat.* 16 35–56. 10.1038/s41575-018-0061-2 30262901

