# Supplementary material for: Marked Seasonal Variation in Structure and Function of Gut Microbiota in Forest and Alpine Musk Deer
Source: Front Microbiol. 2021 Sep 6;12:699797. doi: 10.3389/fmicb.2021.699797 (PMC8450597; doi:10.3389/fmicb.2021.699797)

frontiers

Supplementary Material

Supplementary Figure 1. Rarefaction curves based on Sobs (the observed richness) index and Shannon index at the OTU level of forest musk deer (a, b) and alpine musk deer (c, d). ESpr, LSpr, Sum, Aut and Win represent early spring (T1), late spring (T2), summer (T3), autumn (T4) and winter (T5), respectively. J, A and O represent juvenile individuals, adult individuals and older individuals.

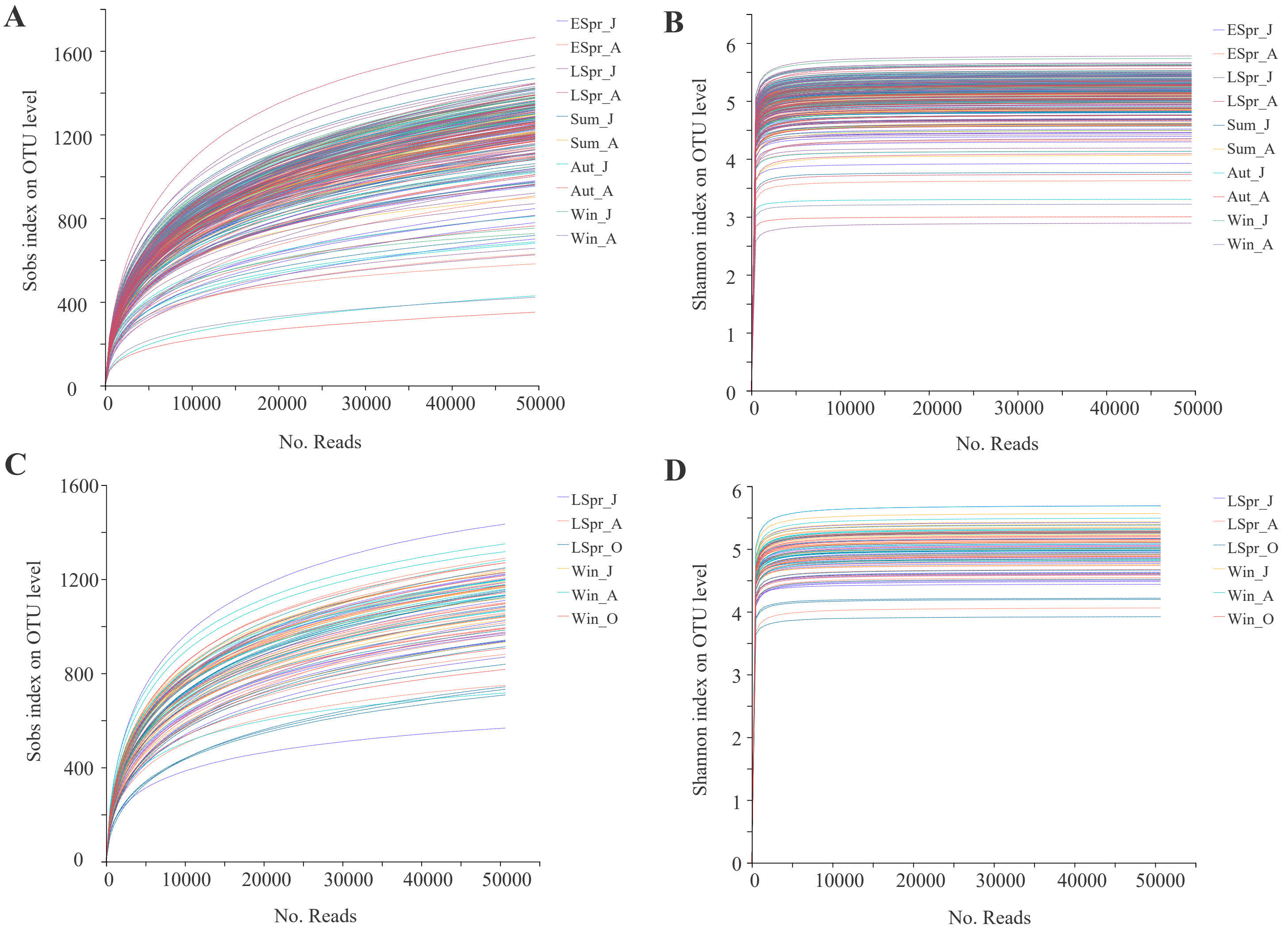

Supplementary Figure 2. PCoA analysis of gut microbial composition between every two seasons in juvenile FMD. Early spring (T1), late spring (T2), summer (T3), autumn (T4) and winter (T5).

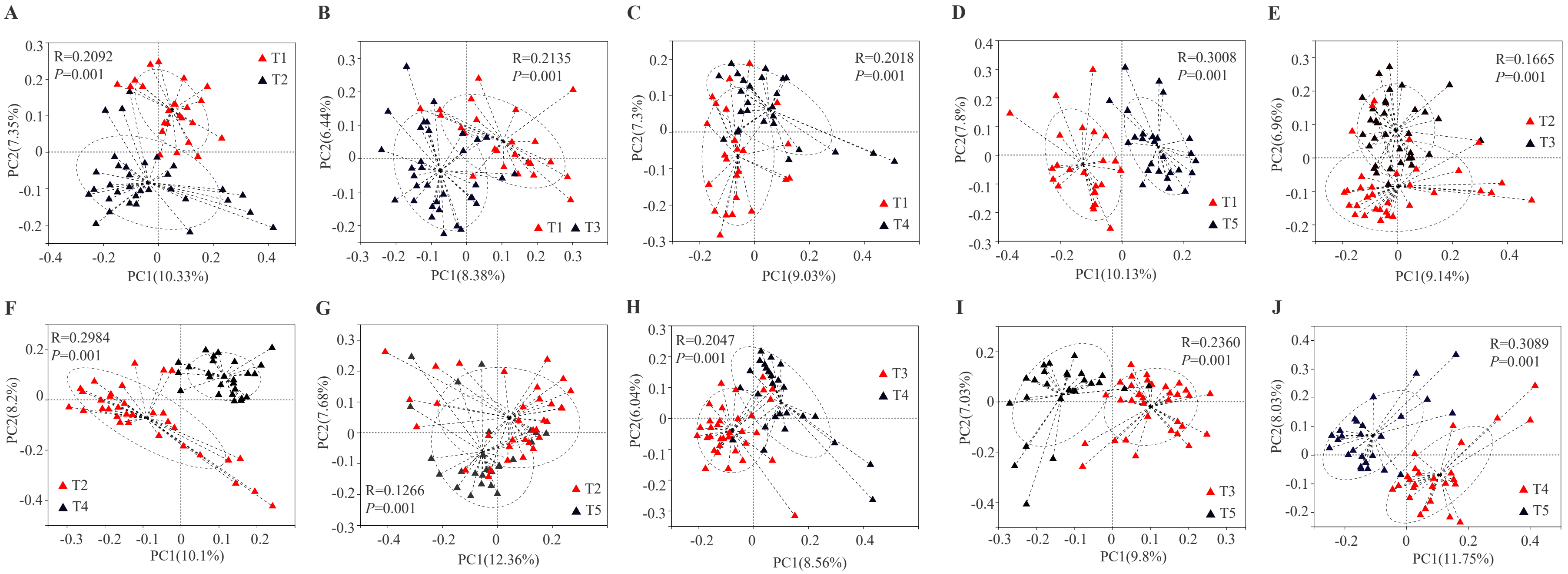

Supplementary Figure 3. PCoA analysis of gut microbial composition between every two seasons in adult FMD. Early spring (T1), late spring (T2), summer (T3), autumn (T4) and winter (T5).

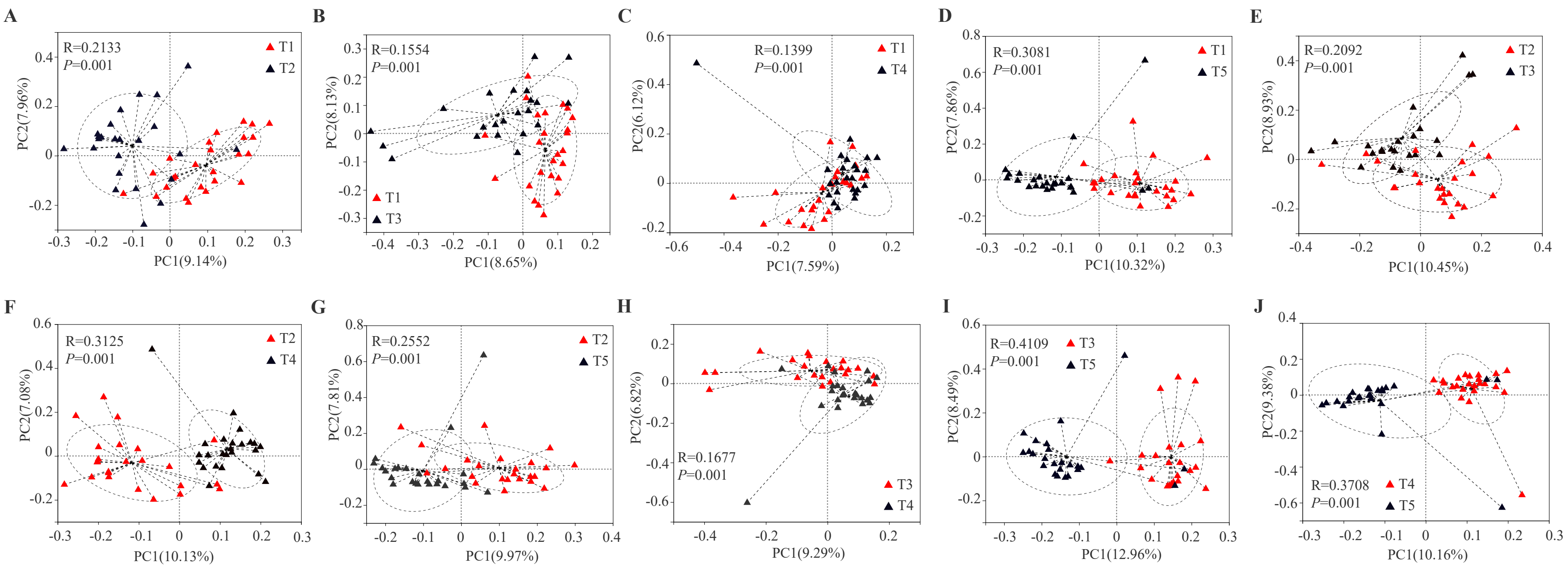

Supplementary Figure 4. Sample dendrogram and trait heatmap. The dendrogram plotted by hierarchical clustering for FMD microbiome composition in 262 included. The heatmap presented below the dendrogram indicates traits for the corresponding samples.

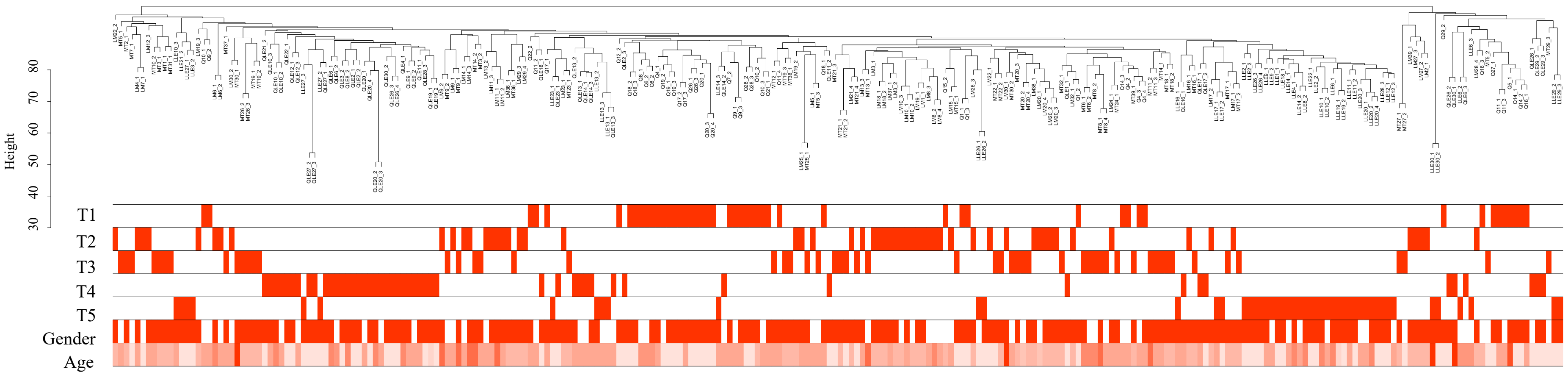

Supplementary Figure 5. Analysis of network topology for various soft-thresholding powers. The top panel shows the scale-free fit index (y-axis) as a function of the soft-thresholding power (x-axis). The bottom panel displays the mean connectivity (degree, y-axis) as a function of the soft-thresholding power (x-axis). Network topology for 1 to 20 soft-thresholding powers. Numbers in the plots indicate the corresponding soft-thresholding powers. The red line indicates scale-free topology fit index at 0.85 as well as blue line indicate mean connectivity at 100. The approximate scale-free topology can be attained at the soft-thresholding power of 8.

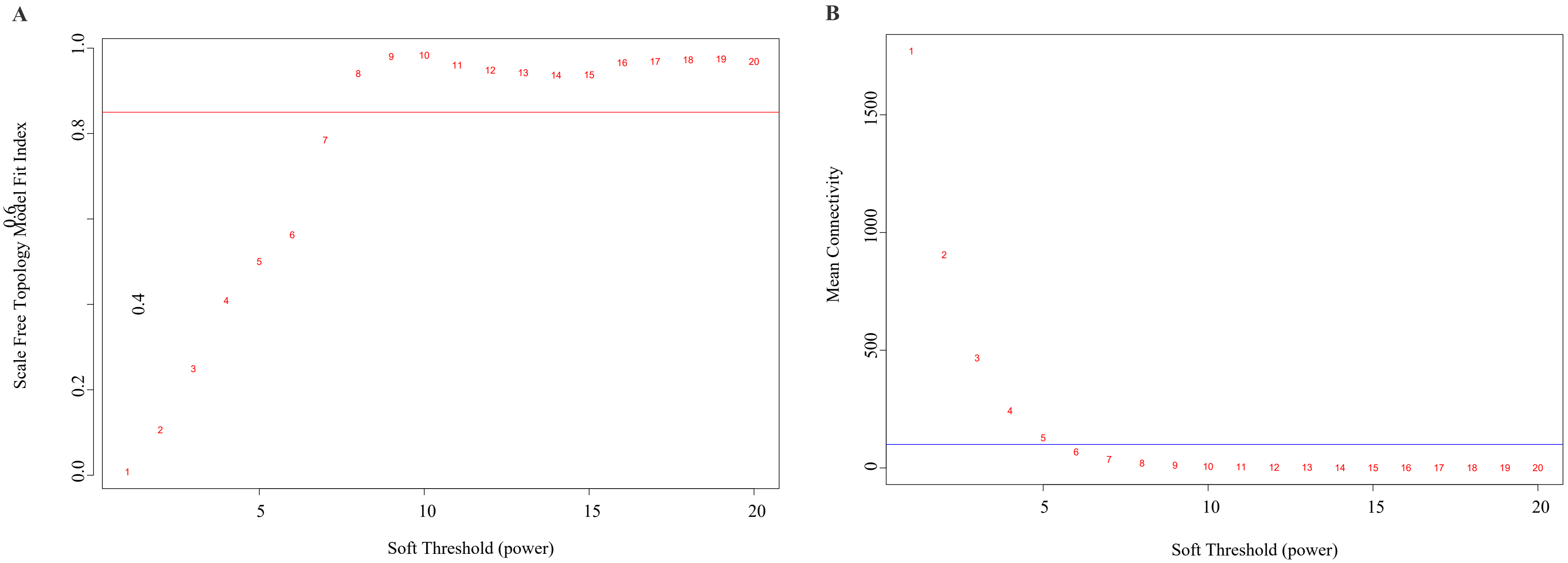

Supplementary Figure 6. Clustering dendrogram of bacterial taxa, with dissimilarity based on topological overlap, together with assigned module colors.

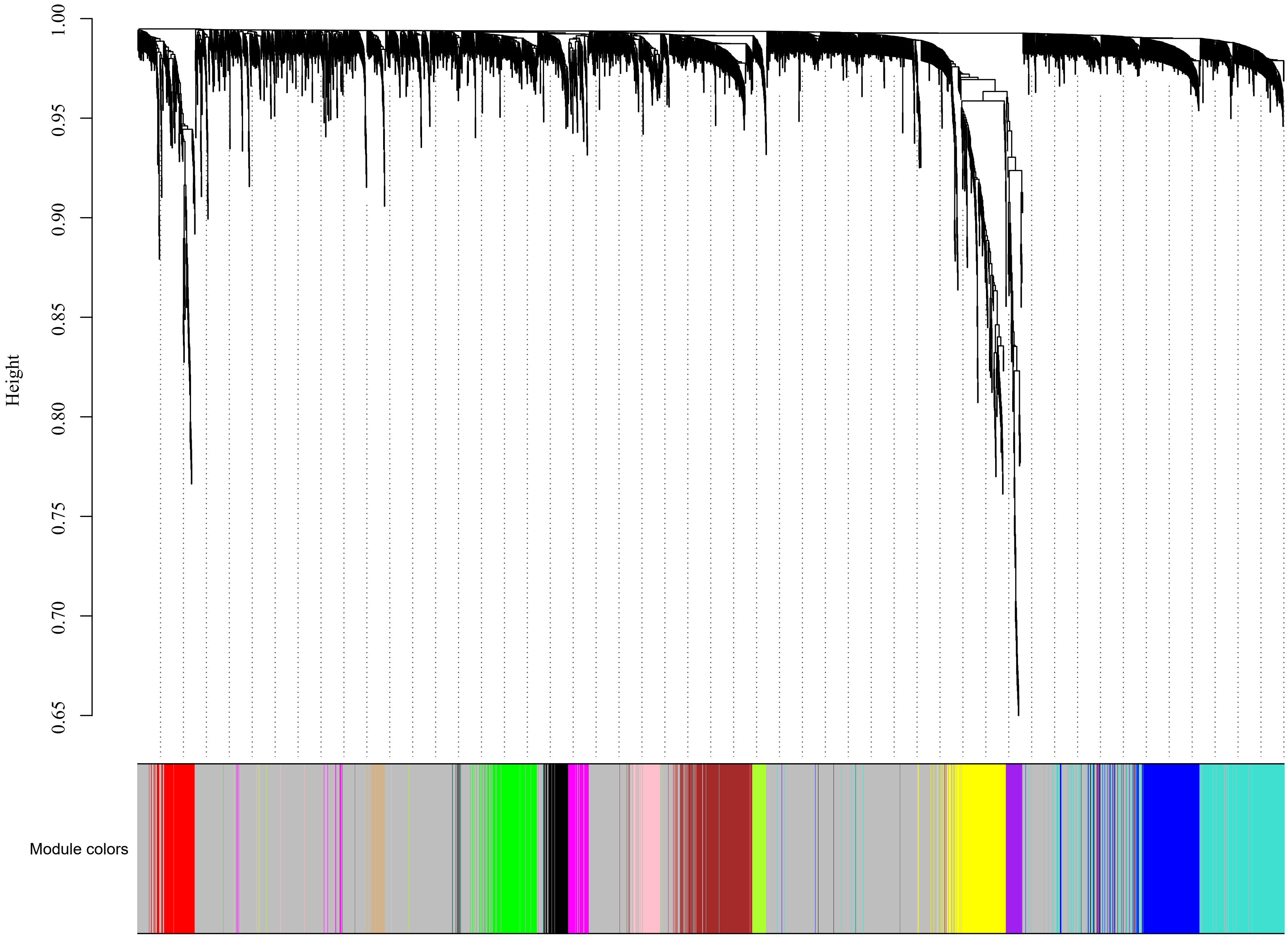

Supplementary Figure 7. Module membership identification using dynamic tree. (A) Clustering dendrogram of bacterial taxa, with dissimilarity based on topological overlap, together with assigned module colors. each leaf, shown as a short vertical line, corresponds to a bacterial taxon. Branches of the dendrogram grouped together densely and interconnected represent highly co-occurring bacterial taxa. Each colored row represents a color-coded module which contains a group of highly co-occurring bacterial taxa. A total of 13 modules were identified. (B) Dendrogram of consensus module eigengenes and heatmap plot of the adjacencies of modules obtained by WGCNA on the consensus correlation. Heatmap plot of the adjacencies of modules. Red represents high adjacency (positive correlation) and blue represents low adjacency (negative correlation).

A

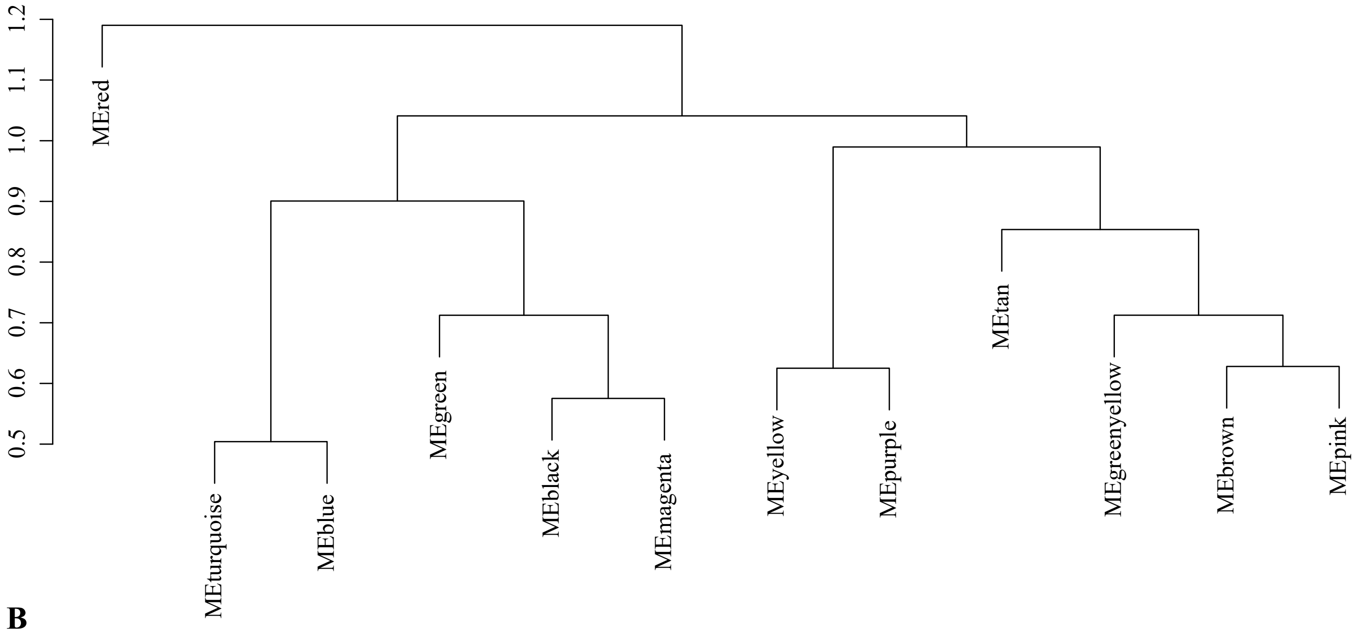

B

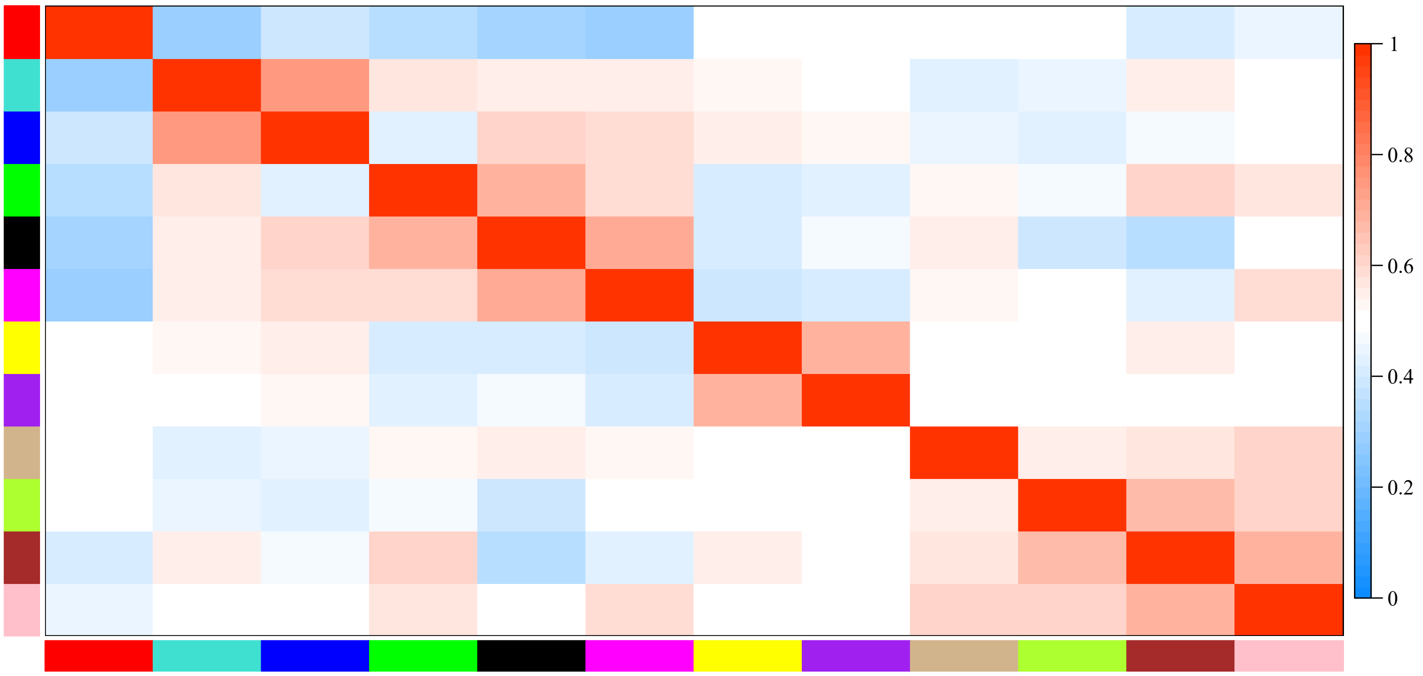

Supplement: Supplementary Figure 1 — Rarefaction curves based on Sobs (the observed richness) index and Shannon index at the OTU level of forest musk deer (A,B) and alpine musk deer (C,D). [file Data_Sheet_1.PDF]
